# Seroprevalence of antibodies to *Plasmodium falciparum* transmission-blocking target proteins Pfs230D1M and Pfs48/45 in Tanzanian populations of diverse malaria transmission intensity

**DOI:** 10.3389/fimmu.2025.1589061

**Published:** 2025-09-11

**Authors:** Charles Mulamba, Wilmina F. Kalinga, Ivanny Mtaka, Linda O. Lazaro, Janeth Kamage, Irene Nkumama, Olukayode G. Odufuwa, Katharina Kreppel, David Mekhaiel, Kazutoyo Miura, Carole A. Long, Ally I. Olotu, Chris Williams

**Affiliations:** ^1^ Interventions and Clinical Trials Department, Ifakara Health Institute, Bagamoyo, Tanzania; ^2^ Nelson Mandela African Institution of Science and Technology, Arusha, Tanzania; ^3^ European Vaccine Initiative, Heidelberg, Germany; ^4^ Transmission-blocking Malaria Vaccine Group, Jenner Institute, University of Oxford, Oxford, United Kingdom; ^5^ Laboratory of Malaria and Vector Research, National Institute of Allergy and Infectious Diseases, National Institutes of Health, Rockville, MD, United States

**Keywords:** malaria transmission, transmission-blocking vaccines, *Plasmodium falciparum*, gametocytes, Tanzania, Pfs25, Pfs230D1M, and Pfs48/45

## Abstract

Transmission-blocking vaccines are among the novel tools under development for malaria control and elimination. Understanding the human immune response to the sexual stages of *Plasmodium falciparum* is essential for progressing transmission-blocking vaccine development. A serosurvey was conducted in Tanzania, from May to August 2022 among 290 participants, consisting of 114 children (5–12 years), 44 adolescents (13–17 years), and 132 adults (18–45 years). The participants were tested for malaria parasites using quantitative polymerase chain reaction, and standardized enzyme-linked immunosorbent assays were performed to detect the presence of IgG antibodies against transmission-blocking target antigens—Pfs230D1M, Pfs48/45, and Pfs25. A set of 10 plasma samples that were reactive to Pfs230DIM and/or Pfs48/45 were tested individually for transmission-reducing activity via standard membrane feeding assays. Of the participants tested, 56% (157/281) had detectable Pfs230D1M antibodies, and 49% (141/290) were positive for Pfs48/45 IgG. Approximately 30% were seropositive for both. However, Pfs25 IgG was not detected in any of the 117 participants tested. The seroprevalence for Pfs230D1M and Pfs48/45 IgG increased significantly with participants’ age, with adults more likely to have antibodies than children: Pfs230D1M (adjusted odds ratio: 3.16, 95% confidence interval: 1.81–5.53, *p*-value ≤ 0.0001) and Pfs45/48 (OR: 3.06, 95% CI: 1.79–5.25, *p* ≤ 0.0001). There was no significant difference in antibody titers for Pfs230D1M and Pfs48/45 antibodies across age groups. A significant transmission-reducing activity was observed in 2/10 participants, who were highly reactive to Pfs230D1M and Pfs48/45. Naturally acquired antibody responses to both full-length Pfs48/45 and Pfs230D1M proteins are prevalent and appeared to be stable, suggesting that semi-immune populations may be ideal to evaluate boosting transmission-blocking vaccine candidates.

## 1 Introduction

Malaria continues to represent a major public health burden globally, leading to 263 million cases and 597,000 deaths, with the heaviest burden carried by sub-Saharan Africa (1–5). Tanzania accounted for 4% of the global malaria deaths reported in 2023 (2), and the Tanzania National Malaria Strategic Plan aims to reduce the malaria burden in moderate- and high-risk areas from 15% to less than 7.5% prevalence in 2025 and to further reduce transmission in low prevalence areas to less than 0.5% *Plasmodium falciparum* parasite rate (pfpr) in 2025 (6). Although the current control methods have led to substantial reductions in malaria morbidity and mortality, progress has slowed, and thus, additional novel tools that may not be impacted by the limitations of the current vector control tools are required (1). Vaccination against malaria is a promising novel tool that is required for a sustainable impact on the disease burden and elimination (5). The licensed malaria vaccines, R21/Matrix-M and RTS,S/AS01, are designed to target the liver stages of the *Plasmodium falciparum* parasites in the human host (7–9). However, the *Plasmodium* parasite has a complex life cycle, involving stages within the human host and the female *Anopheles* mosquito. Interventions that can target other stages of the parasite life cycle (10) are therefore needed. Transmission-blocking vaccines (TBVs) offer a unique approach of interrupting the development of malaria parasites in mosquitoes (11) and could complement the available interventions in controlling and eliminating malaria.

Malaria transmission occurs when at least one male and one female gametocyte are ingested by female *Anopheles* mosquitoes during a blood meal (12). The gametocytes and gametes that egress from red blood cells within the mosquito midgut express specific proteins on their surfaces (13). These surface proteins play an important role in the fertilization, maturation, and colonization of malaria parasites in mosquitoes (14, 15). The parasite surface proteins are categorized into two groups: pre-fertilization and post-fertilization proteins (16, 17), and they form the basis of malaria TBV development. Pfs48/45 and Pfs230 are the most studied *P. falciparum* pre-fertilization antigens and are expressed on both gametocytes and gametes. Pfs230 protein is expressed in both male and female gametocytes and appears on the surface of gametes as a complex with Pfs48/45 (18). Pfs48/45 is highly essential for male and female gamete fusion to form zygotes (19). A proportion of gametocytes die in the human host without being passed on to the mosquitoes, thereby exposing the gametocyte surface antigens to the human immune system. Naturally acquired anti-gametocyte immunity develops after repeated exposure to gametocytes. Based on the production of antibodies against numerous fragments covering Pfs230, Pfs230 domain 1 (Pfs230D1M) has been confirmed as the most robust region for antibody targeting (20). The functional Pfs48/45 antibodies bind to all three domains of Pfs48/45 protein with the strongest functional activity targeting epitopes in domains 1 and 3 (21). The most advanced *P. falciparum* post-fertilisation antigen, Pfs25 (22, 23) is expressed on zygotes and ookinetes, solely in the mosquito vector, but transcripts may occur in circulating gametocytes (24). Though Pfs25 is not a target of naturally acquired immunity in humans, vaccine-induced antibodies have been shown to inhibit oocyst development, which provides a basis for the development of Pfs25 as a TBV (25–27). Transmission-blocking antibodies have been shown to interrupt the fertilization of gametes in the mosquito midgut and the development of transmissible forms of the parasite (28–30).

Naturally acquired antibodies to Pfs230 and Pfs48/45 proteins have been shown to significantly reduce mosquito infection rates by >90% in standard membrane feeding assays (30). However, there is limited knowledge on the prevalence and variation of these antibodies in different malaria endemic settings and how they relate to factors that influence transmission, including age, parasitemia, and transmission intensity, among other factors. Assessment of naturally induced anti-gametocyte responses to parasite target proteins is of paramount importance for progressing the development of transmission-blocking vaccines, especially in malaria-endemic settings. Here, we determined the seroprevalence, levels, and potential functionality of naturally acquired antibodies to *P. falciparum* transmission-blocking target antigens Pfs230D1M and full-length Pfs48/45, as a baseline for a phase 1 clinical evaluation of a transmission-blocking vaccine candidate, Pfs25-IMX313/Matrix-M, in Bagamoyo, Tanzania.

## 2 Methods

### 2.1 Study area

The study was conducted in Bagamoyo District, located in the coastal region of Tanzania. The district represents heterogeneous malaria transmission, with malaria prevalence in the general population estimated to be between 7% and 39% (31, 32). Five sites in Bagamoyo District with varying malaria transmission intensities categorized following the guidelines of the U.S. President’s Malaria Initiative (PMI) (32) were surveyed: Bagamoyo township, Fukayosi, and Yombo with low to moderate (5% to 30% malaria infection prevalence) and Wami-Mkoko and Miono with high malaria transmission intensity (>30% malaria infection prevalence) (37). The malaria transmission intensity is usually high during and after the long rainy season (33), which usually occurs from March to June. The majority of the reported cases are caused by *P. falciparum*, but other *Plasmodium* species have also been reported (31, 34–36). Reported vector control practices employed in the study area are bed nets with 69% coverage (households with one bed net for every two persons) and house modification with 65% of the households with screened windows and 13% with closed eaves (38). There was no mass drug administration done in the study area.

### 2.2 Study design and population

A serosurvey was conducted between May and August 2022. A total number of 290 participants aged 5–45 years, without malaria clinical symptoms, were enrolled using simple random sampling from primary schools, peripheral dispensaries, and community-based malaria testing camps. Participants were invited for antibody and malaria testing following mass sensitization by village health teams. Among the 290 recruited participants, Pfs48/45 IgG was tested in all, Pfs230D1M and Pfs48/45 IgG in 281, and all three antigens (Pfs48/45, Pfs230D1M, Pfs25) in 117. Antibody persistence was assessed in 57 participants, sampled five times from July 2022 to March 2023, following baseline sampling in June 2022. Antibody functionality was assessed in 10 of the 57 participants via standard membrane feeding assays.

### 2.3 Blood sample collection and preservation

Blood samples were collected into 2.0-mL tubes containing ethylenediaminetetraacetic acid (EDTA). A volume of 200 µL was immediately preserved in 600 µL of 1× DNA/RNA Shield™ (Zymo Research, Irvine, USA) for nucleic acid extraction to detect circulating malaria parasites in each participant, using quantitative polymerase chain reaction (qPCR). A volume of 300 µL of the collected plasma was used for detecting total IgG antibodies against Pfs230D1M, Pfs48/45, and Pfs25 antigens. The remainder of the 2-mL blood sample was reserved for determining antibody functionality via standard membrane feeding assays.

### 2.4 Recombinant proteins

Production of both full-length Pfs48/45 (39) and Pfs25 (40) has been described previously (39, 40). A truncated recombinant version of Pfs230, termed Pfs230D1M (41), was based on the full-length 3D7 sequence (GenBank™ accession number XP_001349600.1), comprising residues Ser^542^ to Gly^736^ with a single Asn to Gln mutation at position 585 to remove an N-glycosylation site. Pfs230D1M was expressed in the laboratory as a secreted protein using the pPIC9K vector (Invitrogen, California, USA) in *Pichia pastoris* yeast (Invitrogen). All proteins contained a C-terminal EPEA motif to allow for the use of C-tag-based affinity chromatography (Thermo Fisher Scientific, UK) for the initial capture, followed by a gel filtration polishing step on a HiLoad 16/600 Superdex 200 PG column (Cytiva, Uppsala, Sweden).

### 2.5 The standardized enzyme-linked immunosorbent assay

The enzyme-linked immunosorbent assay (ELISA) was performed on plasma samples to detect and measure levels of total IgG to recombinant Pfs48/45, Pfs230D1M, and Pfs25 antigens following the protocol described previously (42). Briefly, ELISA plate wells were coated overnight with 50 μL of either Pfs48/45, Pfs230D1M, or Pfs25 recombinant proteins at a concentration of 2 μg/mL. The plates were blocked with StartingBlock™ T20 (PBS) Blocking Buffer (Thermo Fisher Scientific, UK) for 1 h before 50 μL of the diluted test plasma samples were added into the ELISA microtiter plate in triplicate. The plates were then incubated for 2 h at room temperature before a detecting antibody [goat anti-human IgG (γ-chain) conjugated to alkaline phosphatase] was added. The plates were incubated at room temperature for 1 h, and 100 µL of the pNPP with diethanolamine substrate was added to each well to produce fluorescence. The substrate was left to develop for 25 min, and the absorbance at 405 nm was read using the BioTek microplate reader (ELx808) and Gen5 software (v3.04). Between each incubation step, plates were washed six times with 1× PBS + 0.05% Tween 20. The ELISAs were performed using a standard curve and internal positive controls from reference samples. Known anti-Pfs25 serum from De Graaf et al. (43) was used as a reference positive control for Pfs25 ELISAs. The Pfs230D1M and Pfs48/45 reference plasmas from 20 donors (10 donors for each) were prepared separately as described previously (42). Briefly, Pfs230D1M or Pfs48/45 reactive plasma was pooled and optimized at a 1:800 dilution in StartingBlock™ T20 (PBS) Blocking Buffer. For each plate, a total of six wells were incubated with a positive control and negative control sera from unexposed United Kingdom (UK) donors. Wells containing no test sera were used to deduct background reactivity from each sample. A seropositivity cutoff point was set at an OD above the mean + 2SDs of a panel of UK donors. A standard curve and Gen5 ELISA software v3.04 (BioTek, UK) were used to convert the OD405 of individual test samples into arbitrary units (AU).

#### 2.5.1 Antibody avidity determination by ELISA

Sodium thiocyanate (NaSCN, a chaotropic agent)-based avidity assays were conducted to estimate the avidity of antibody responses before the assessment of antibody functionality according to a protocol previously described (44). Briefly, each plasma was diluted based on standardized ELISA readouts to reach an optical density (OD) of 1. The plasma was added into ELISA plate wells coated overnight with 50 µL/well of 2 µg/mL Pfs230D1M or Pfs48/45 protein. Concentrations of the NaSCN chaotropic agent, ranging from 0 to 7 M, were added in increasing concentration down the plate. The plates were incubated at RT for 15 min, and a detecting secondary antibody was added, followed by pNPP with diethanolamine substrate (100 µL/well). The plates were allowed to develop before being read at 405 nm OD on BioTek ELx808 microplate reader with Gen5 software. Avidity index (mol/L) was determined as the concentration of NaSCN which gave 50% of OD values compared to no NaSCN. The binding of antibodies with lesser avidity to the antigen is disrupted at concentrations of NaSCN lower than for antibodies with greater avidity.

### 2.6 *Ex vivo* functionality of Pfs230D1M and/or Pfs48/45-positive IgG

The NF54 laboratory strain of *P. falciparum* gametocytes was used to evaluate the functional activity of Pfs230D1M and Pfs48/45 antibodies. Gametocyte cultures were maintained according to a previously published protocol (45). Briefly, cultures were initiated at an asexual parasitemia of 0.15%–0.2% and hematocrit of 5% in 10 mL complete medium (RPMI-1640 with 6 g/L of HEPES, 50 mg/L of hypoxanthine, 2.5 g/L of sodium bicarbonate, and 10% human serum). Cultures were maintained for 16 days under controlled gas conditions (90% nitrogen, 5% carbon dioxide, 5% oxygen), with daily media changes. More than three identical replicate cultures were maintained. For each experiment, at least two replicates were pooled based on stage V gametocytemia and exflagellation levels. The culture medium was replaced with normal human serum, normal red blood cells (RBCs) at a stage V gametocytemia of approximately 0.15%, and 50% hematocrit (1 mL of serum:1 mL of RBCs). The male-to-female gametocyte ratio was stabilized at 1:2–3. The human serum and RBCs used were obtained from Interstate Blood Bank, Inc., USA.

The ability of total IgG positive for Pfs230D1M and/or Pfs48/45 antibodies to reduce *P. falciparum* infections in mosquitoes was assessed using standard membrane feeding assays (SMFAs) as previously described in Miura et al. (45).

Total IgG from individual samples was purified using Protein G affinity chromatography and reconstituted to final concentrations of 40 mg/mL in PBS. The purified human IgG (at 10 mg/mL) was then mixed with NF54 *P. falciparum* gametocytes at 30°C and immediately fed to 4–6-day-old laboratory-reared *Anopheles stephensi* mosquitoes via a Parafilm^®^ membrane. Mouse monoclonal antibody 4B7 at a concentration of 93.8 μg/mL was used as a positive control. The negative control was either a purified IgG (10 mg/mL) from pooled malaria naive human sera (SMFA-1) or that (0.75 mg/mL) from a pooled normal mouse serum (SMFA-2). The mosquito midguts were dissected 8 days post-feeding and examined microscopically to assess oocyst intensities in the test and control assays. A total of 20 blood-fed mosquitoes per volunteer were dissected for every SMFA round. Transmission-reducing activity (TRA), a measure of percentage (%) reduction in oocyst intensity, was calculated relative to the respective control IgG tested in the same assay. TRA was calculated as follows: 100 × {1 − (mean number of oocysts in the test IgG)/(mean number of oocysts in the control IgG)} (46). The average oocysts and % infectivity for the negative control IgGs were 34 oocysts per mosquito and 100% infectivity in SMFA-1 and 19 oocysts and 98% in SMFA-2.

### 2.7 Total parasites and gametocyte detection by RT-qPCR

Total parasitemia was determined by qPCR performed on the blood samples preserved in DNA/RNA Shield™ (47, 48), using CFX96 real-time qPCR thermocycler as detailed in Mulamba et al. (37). Genomic DNA was extracted from *Plasmodium*-positive blood samples using a Quick-DNA Miniprep Plus Kit (Zymo Research, USA) and eluted in 50 µL of elution buffer. The qPCR targeting the *Pan-Plasmodium* 18S rRNA and *P. falciparum*-specific varATS sequences was performed. This approach enhances both the specificity and sensitivity for detecting *P. falciparum* and non-*falciparum Plasmodium* species.

To determine gametocytemia, a combination of two independent *Plasmodium* targets—the female gametocyte-specific marker (CCp4) and the male gametocyte-specific marker (*Pf*MGET) from Meerstein−Kessel et al.—was used (49). Ribonucleic acid (RNA) was extracted from all qPCR-positive samples, using the Quick-RNA™ MiniPrep Plus kit (Zymo Research). The protocol was adapted according to the manufacturer’s instructions as previously described (31). *Plasmodium falciparum* gametocytes were detected using a multiplex qRT-PCR assay described previously by Meerstein−Kessel et al. (49) as detailed in Mulamba et al. (37).

### 2.8 Statistical analysis

Data were recorded in Excel (Microsoft, 2016) and analyzed using Stata version 16 (50) and GraphPad Prism version 10 (GraphPad Software Inc., California, USA). Descriptive analysis was conducted on the demographic characteristics of the participants and the status (negative or positive) in relation to gametocyte and antibody presence using proportions and their respective 95% confidence intervals (CIs). The antibody responses were descriptively analyzed using geometric mean (95% confidence interval) and presented in tables. Pearson correlation coefficient (PCC) was used to assess the correlation between the log of parasite density and the log of antibody titers and the correlation between the titers of Pfs230D1M and Pfs48/45 antibodies. Binary logistic regression was used to estimate the association between antibody titers and the following factors: age groups (5–12, 13–17, and 18–45 years), sex (male and female), and location (low and high transmission) of the participants. The likelihood ratio test was also performed to assess the influence of each of the variables in the multivariate model. For the SMFA results, a zero-inflated negative binomial model was used (45). Binary logistic regression was also employed to assess the association between parasite positivity in relation to antibody seropositivity, with demographic characteristics of age groups, sex, and location of the participants adjusted for fixed effects. Furthermore, the change in Pfs230D1M and Pfs48/45 antibodies over time was explored using mixed-effect gamma regression, given the non-normal distribution of the data, and unique identification adjusted for random effects for the unadjusted models. The adjusted models included age groups, sex, and location of the participants as fixed effects.

## 3 Results

### 3.1 Study participants

Of the 290 participants recruited, 114 (39%) were children (5–12 years), 44 (15%) adolescents (13–17 years), and 132 (46%) adults (18–45 years). The average age (years) in each age group was 9, 15, and 31 years, for children, adolescents, and adults, respectively. The proportion of female participants was 51%. Recruitment varied between low [124 (43%)] and high [166 (57%)] malaria transmission sites (**Table 1**).

**Table 1:** The study participants’ demographic characteristics.

Age group	Low–moderate transmission			High transmission		
	Female	Male	Subtotal	Female	Male	Subtotal
	*n* (%)	*n* (%)	*n* (%)	*n* (%)	*n* (%)	*n* (%)
Children	25 (42.4)	18 (27.7)	43 (34.7)	42 (46.7)	29 (38.2)	71 (42.8)
Adolescent	9 (15.3)	16 (24.6)	25 (20.2)	6 (6.7)	13 (17.1)	19 (11.5)
Adult	25 (42.4)	31 (47.7)	56 (45.2)	42 (46.7)	34 (44.7)	76 (45.8)
Subtotal	59 (100)	65 (100)	124 (100)	90 (100)	76 (100)	166 (100)

### 3.2 Prevalence of anti-Pfs230D1M and anti-Pfs48/45 antibodies

**Figure 1:**
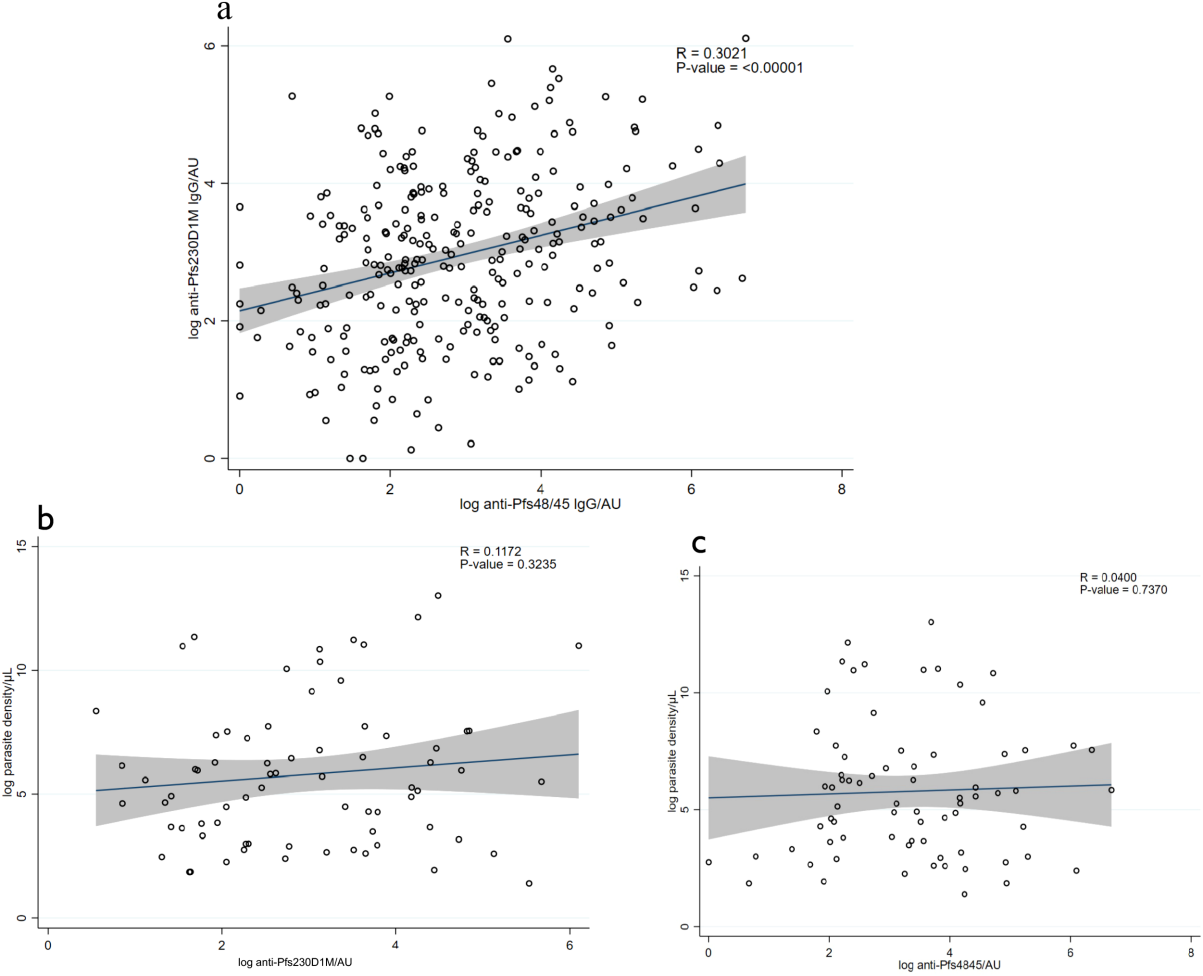
Correlation between antibody levels and parasite density. **(A)** Correlation between anti-Pfs230DIM antibody levels and anti-Pfs48/45 levels in the figure. **(B)** Correlation between anti-Pfs230D1M antibody levels and malaria parasite density. **(C)** Correlation between anti-Pfs48/45 levels and parasite density. The solid line shows the predicted mean from the linear regression, and the shaded area is the confidence interval.

Overall, 56% (157/281) and 49% (141/290) of the participants were categorized as seropositive for anti-Pfs230D1M and anti-Pfs48/45 IgG, respectively. Approximately 30% (86/281) of the participants were seropositive for both Pfs230D1M and anti-Pfs48/45 IgG (**Table 2**). There was no anti-Pfs25 IgG detected among the 117 participants tested. The seroprevalence for anti-Pfs230D1M and anti-Pfs48/45 IgG increased significantly with participants’ age, with adults more likely to have antibodies than children—Pfs230D1M [adjusted OR 3.18 (95% CI: 1.85–5.57), *p* ≤ 0.0001] and Pfs45/48 [OR 3.18 (95% CI: 1.83–5.29), *p* ≤ 0.0001]—reflecting cumulative exposure to infection (**Table 2**). There was a significant positive correlation between titers for anti-Pfs48/45 and anti-Pfs230D1M IgG (*R*-value: 0.3021, *p* = 0.00001; **Figure 1A**).

**Table 2:** Pfs230D1M and Pfs48/45 seropositivity in relation to the demographic characteristics.

Demographics	Pfs230D1M				Pfs48/45				Combination of Pfs230D1M and Pfs48/45			
	*n* ^a^	% (*n* ^a^/*N* ^a^)	GM (95% CI)	aOR (95% CI) *p*-value	*n* ^b^	% (*n* ^b/^/*N* ^b^)	GM (95% CI)	aOR (95% CI) *p*-value	*n* ^c^	% (*n* ^c^/*N* ^c^)	GM (95% CI)	aOR (95% CI) *p*-value
Overall	157	55.9	3.7 (3.6–3.9)	–	141	48.6	3.9 (3.8–4.1)	–	86	30.6	6.5 (6.2–6.7)	–
Age group												
Children	44	41.9	3.5 (3.3–3.7)	Ref	42	36.8	3.9 (3.5–4.2)	Ref	21	20.0	6.1 (5.7–6.4)	Ref
Adolescent	20	45.5	3.8 (3.4–4.1)	1.05 (0.51–2.19) 0.889	14	31.8	3.9 (3.4–4.5)	0.76 (0.35–1.64) 0.489	7	15.9	6.2 (5.6–6.7)	0.76 (0.29–1.98) 0.580
Adult	93	70.5	3.8 (3.7–4.0)	2.84 (1.65–4.87) <0.0001	85	64.4	4.0 (3.8–4.1)	3.15 (1.85–5.37) <0.0001	58	43.9	6.8 (6.5–7.1)	3.43 (1.90–6.20) <0.0001
Sex												
Female	79	54.5	3.7 (3.5–3.8)	Ref	64	42.9	3.8 (3.6–4.0)	Ref	37	25.5	6.2 (5.9–6.5)	Ref
Male	78	57.4	3.8 (3.6–4.0)	1.07 (0.65–1.77) 0.777	77	54.6	4.1 (3.9–4.3)	1.77 (1.07–2.91) 0.025	49	36.0	6.8 (6.4–7.1)	1.72 (1.01–2.95) 0.047
Location												
Low−moderate	72	62.6	3.7 (3.6–3.9)	Ref	52	41.9	3.9 (3.6–4.1)	Ref	34	29.6	6.2 (5.9–6.6)	Ref
High	85	51.2	3.7 (3.6–3.9)	0.59 (0.36–0.99) 0.045	89	53.6	4.0 (3.8–4.2)	1.70 (1.03–2.81) 0.038	52	31.3	6.6 (6.3–6.9)	1.25 (0.73 – 2.16) 0.419

No anti-Pfs25 IgG was found in the study; 105/114 children enrolled in the study were tested for both Pfs230D1M and Pfs48/45 IgG.

*N* ^a^, total number of participants who tested for Pfs230D1M IgG; *n* ^a^, number of participants who tested positive for anti-Pfs230D1M IgG; *n* ^b^, number of participants who tested positive for Pfs48/45 IgG; *N* ^b^, total number of participants who tested for Pfs48/45 IgG; *n* ^c^, number of participants who tested positive for both Pfs230D1M IgG and Pfs48/45 IgG; *N* ^c^, number of participants tested for both Pfs230 IgG and Pfs48/45 IgG; GM, geometric mean of antibody titers; 95% CI, 95% confidence interval.

### 3.3 Parasite positivity in relation to antibody seropositivity

The total parasite and gametocyte positivity was 23.5% and 5.6%, respectively, and has already been published elsewhere (37). There was a significant association between Pfs48/45 and parasite positivity (**Table 3**).

**Table 3:** The association between antibody seropositivity and parasite positivity.

Outcome	Had antibodies and parasites	Descriptive	Unadjusted		Adjusted for covariates	
		Prevalence % (*n*/*N*)	Odds ratio	*p*-value	Odds ratio	*p*-value
Pfs230D1M	No	49.3 (36/124)	1.00		1.00	
	Yes	50.7 (37/157)	0.75 (0.44–1.29)	0.300	0.97 (0.54–1.75)	0.917
Pfs48/45	No	39.7 (29/149)	1.00		1.00	
	Yes	60.3 (44/141)	1.88 (1.09–3.22)	0.022	1.90 (1.04–3.50)	0.038

Covariates adjusted for fixed effect: age groups (5–12, 13–17, and 18–45 years), sex, and location.

#### 3.3.1 Correlation between antibody levels and parasite density

There was no correlation between the antibody titers and total parasite density, as indicated in **Figures 1B, C**: Pfs230D1M antibody titers and parasite density (*R*-value: 0.1172, *p*-value = 0.3235) and anti-Pfs48/45 titers and parasite density (*R*-value: 0.0451; *p*-value = 0.7046).

### 3.4 Persistence of Pfs230D1M and Pfs48/45 antibodies over time

**Figure 2:**
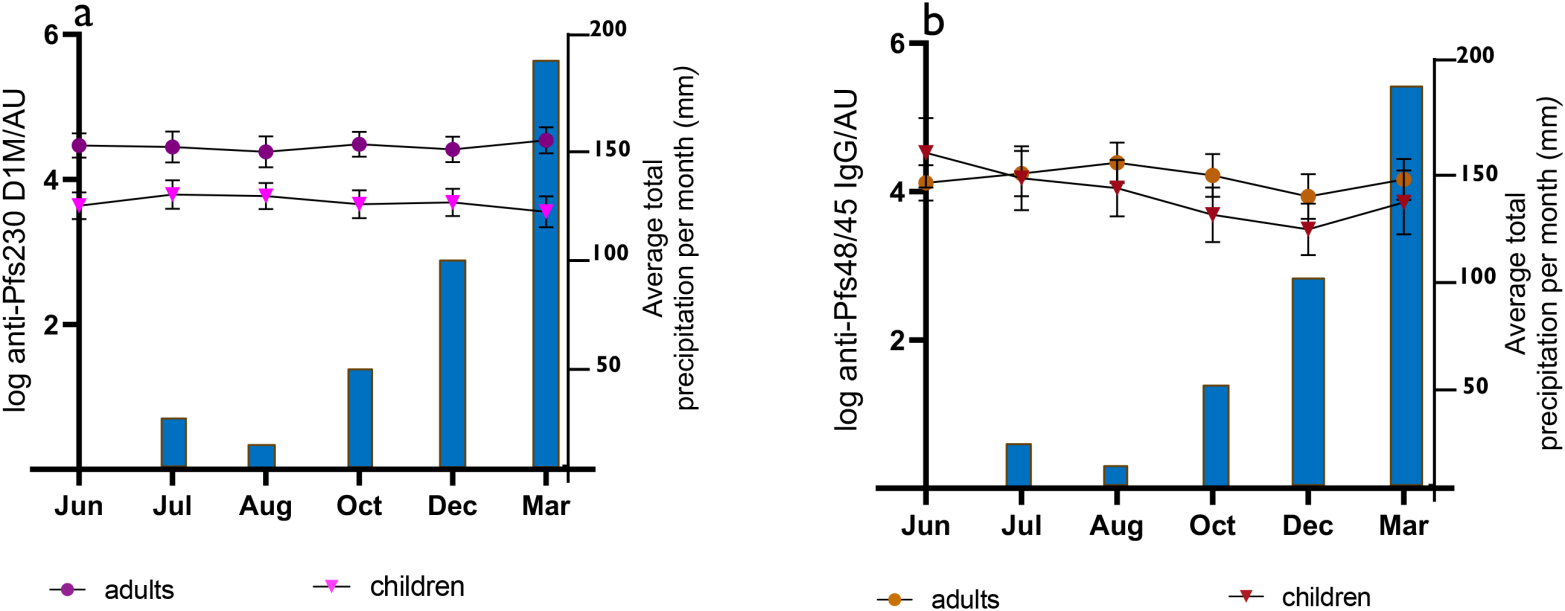
Geometric mean anti-Pfs230D1M and anti-Pfs48/45 IgG titer in seropositive adults and children over a 10-month-period: **(A)** anti-Pfs230D1M total IgG responses; *n* = 31 (8 children and 23 adults); **(B)** anti-Pfs48/45 total IgG responses; *n* = 26 (9 children and 17 adults), and antibody responses are shown on the left *y*-axes. The error bars indicate the lowest and highest antibody titer. Average precipitation at each time point is indicated by blue bars (right *y*-axes). The sampling time points were between June 2022 and March 2023.

To assess the persistence of anti-Pfs230D1M and anti-Pfs48/45 over time, a group of 33 adults and 24 children, conveniently selected from high and low transmission areas, were sampled at five different time points spanning over a period of 10 months in addition to baseline (June 2022) sampling. Of the 57 participants included in the longitudinal analysis, 39 originated from high transmission areas and 18 from low transmission areas (**Supplementary Table S1**). We were unable to sample adolescents during the 10-month period. Seropositive participants at baseline remained seropositive at each of the later time points, and their geometric mean antibody responses are shown in **Figure 2**. There was no significant difference in Pfs230D1M antibody responses over time, while a significant decrease in titers for Pfs48/45 antibodies between August 2022 and March 2023 was shown. Participants who were seronegative at baseline remained seronegative at all time points and were not included in the analysis.

### 3.5 Small proportion of participants positive for Pfs230D1M and Pfs48/45 antibodies showed significant TRA

**Figure 3:**
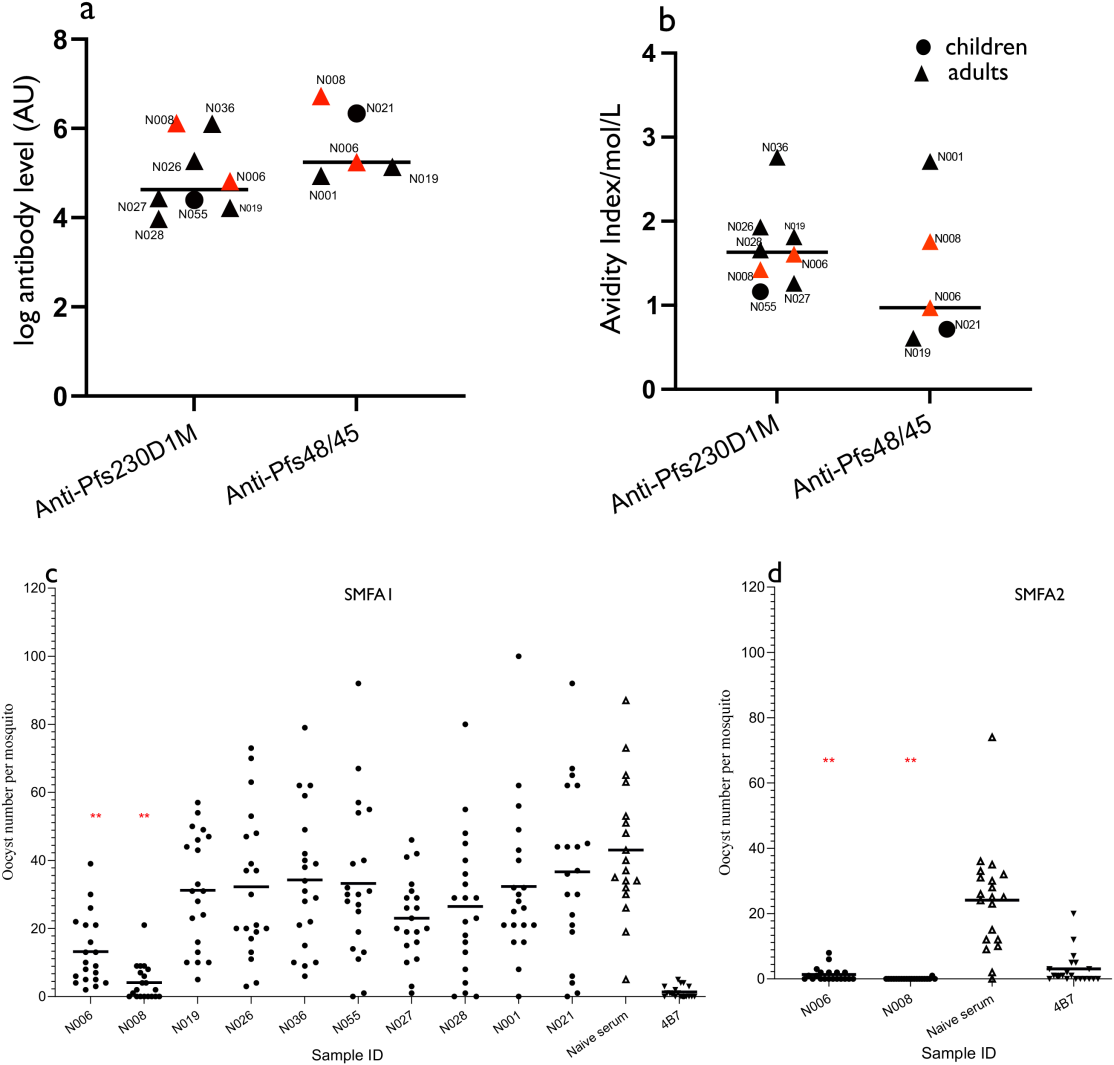
Antibody levels, antibody avidity, and oocyst intensity in participants assessed for antibody functionality. **(A)** Pfs230D1M and Pfs48/45 individual and median Pfs230D1M and Pfs48/45 antibody responses. **(B)** Individual and median avidity indices of total Pfs230D1M and Pfs48/45 IgG. Plasma samples were tested individually, and increasing concentrations of NaSCN (0, 1, 2, 3,4,5, 6, 7, and 8 M) were used prior to the development of the ELISA reaction. Results for both ID N006 and N008 are in red. IC50 = concentration of NaSCN needed to reduce antibodies by 50%. **(C, D)** Oocyst counts following SMFA with purified IgGs from Pfs230D1M and/or Pfs48/45-positive plasmas. Purified IgG (10 mg/mL) was mixed with *P. falciparum* NF54 cultured gametocytes and fed to mosquitoes (*n* = 20 per test assay), which were dissected 7 days post-feeding to determine oocyst intensity. Purified IgG from a pool of naive sera was used as negative control. Monoclonal Pfs25 antibodies (4B7) was used as the positive control. The *asterisk* means statistically significant difference (*p*-value < 0.05) between the negative control and test samples. All 10 samples were tested in SMFA1 (**C**), and only two samples were tested in SMFA2 (**D**).

Purified IgGs from baseline plasma samples from 10 participants reactive to Pfs230D1M and/or Pfs48/45 antigens by ELISA were individually tested for TRA via a single SMFA. The 10 participants selected were from high transmission areas and included two children and eight adults, for whom adequate plasma samples had been collected. Individual antibody responses and avidity indices of the 10 participants are shown in **Figures 3A, B**. Three participants (N006, N008, and N019) were reactive to both Pfs230D1M and Pfs48/45 antigens. Two participants (N001 and N021) were reactive to the Pfs48/45 antigen only. Five participants (N026, N055, N027, N028, and N036) were reactive to Pfs230D1M antigen only. Only two participants (N006, N008), who were adults, demonstrated significant TRA (**Figure 3C**; **Supplementary Table S2**). Independent SMFA performed for N006 and N008 confirmed their significant inhibitory activity (**Figure 3D**).

## 4 Discussion

The present study is the first to determine the prevalence of naturally acquired antibodies to Pfs230D1M and Pfs48/45 transmission-blocking parasite proteins in Bagamoyo. These observations need to be taken into consideration in the planning of vaccine trials that have to account for pre-existing immunity and potential immune boosting following natural antigen exposure. We found strong evidence of naturally acquired antibody responses against *P. falciparum* gametocyte antigens—Pfs230D1M and Pfs48/45—as previously reported (28, 33, 51, 52). All the participants tested for anti-Pfs25-specific IgG were seronegative, most likely because Pfs25 transcripts are thought to be translated only in mosquito midgut post-blood ingestion.

The higher seroprevalence of Pfs230D1M and Pfs48/45 antibodies in adults compared to children is most likely due to repeated exposure to gametocytes over the years, as previously observed (28, 51). There was no significant difference in anti-Pfs48/45 and anti Pfs230D1M titers detected in children, adolescents, and adults, but a significant difference was observed in seroprevalence between areas of low and high transmission. This indicates a potential role for recent parasite exposure in the presence of Pfs48/45-specific immunity.

A large proportion of participants demonstrated positive anti-Pfs230D1M or anti-Pfs48/45 IgG responses in the absence of gametocytes or parasite infection at the time of sample collections. The detected anti-gametocyte responses were most likely resulting from immune responses from previous gametocyte exposure prior to sampling. This observation suggests that antibodies may serve as markers of gametocyte exposure, rather than biomarkers of active gametocytemia. However, 30%–40% of adults were ELISA-negative for Pfs230D1M or Pfs48/45 antigens, but it is highly likely that they have been infected multiple times. Concurrent gametocytes may reflect the development of anti-gametocyte responses, but a direct association may not always be observed (28, 51). Anti-gametocyte antibodies induced before sample collection may persist for several weeks after gametocyte clearance. On the other hand, antibody induction/boosting to circulating gametocytes at the time of sampling may take longer, e.g., after their death/destruction and subsequent clearance (33, 51).

The 10-month period over which anti-gametocyte responses were measured coincided with the end of a long rainy season and the start of the next rainy season. Data from this period indicated that both anti-Pfs230D1M and anti-Pfs48/45 IgG responses were stable, although this study did not answer whether the stable IgG responses were due to longevity of antibody response, repeat gametocyte exposures, or a mixture of both. More detailed longitudinal studies should be undertaken with a bigger sample size and larger age range to firmly establish the age dependency of anti-gametocyte malaria immunity as well as provide more evidence on the longevity of these responses.

The lack of significant inhibition of oocyst intensity by individuals who were reactive to either Pfs230 or Pfs48/45 antigens suggests that the antibodies could be binding to non-blocking epitopes or that the antibody levels were not sufficient to affect oocyst reduction in the mosquitoes. The observation of high transmission-reducing activity in the two samples reactive to both Pfs48/45 and Pfs230D1M antigens is similar to reports from previous field studies (33), and the observed TRA may be attributed to an additive or synergistic effect of antibodies to two different antigens. However, it is also possible that these two individuals were reactive to an unknown transmission-blocking antigen, and further investigations are required to fully understand the immune responses promoting the observed TRA in the two participants.

Several limitations were encountered in this study; the participants were recruited from only five locations, which were easily accessible, and this may have restricted the study area and sample size. In addition, we were unable to perform antibody depletion assays to ascertain if the TRA observed in the two participants was promoted by Pfs48/45 or Pfs230D1M IgG. Despite these limitations, the prevalence of naturally acquired antibodies to Pfs48/45 and Pfs230D1M in this study bodes well for the potential of vaccine-induced immunity against these antigens as suggested by other studies (53, 54). A recent phase 1 trial of Pfs48/45 and Pfs230 vaccine candidates in Burkina Faso found that vaccine-specific responses boosted pre-existing immunity against respective antigens (55), which contrasts prior findings that exposure to *P. falciparum* might diminish subsequent boosting by vaccination (56). These experiences indicate the need for transmission-blocking vaccine evaluation in malaria-endemic settings to take into account naturally acquired immune responses to the sexual-stage vaccine antigens. Naturally acquired antibody responses to both Pfs48/45 and Pfs230D1M proteins are highly prevalent and appear to be stable, suggesting that semi-immune populations may be ideal to evaluate boosting transmission-blocking immunity by transmission-blocking vaccine candidates.

## Data Availability

The original contributions presented in the study are included in the article/**Supplementary Material**. Further inquiries can be directed to the corresponding author.
