# Supplementary material for: Seroprevalence of antibodies to Plasmodium falciparum transmission-blocking target proteins Pfs230D1M and Pfs48/45 in Tanzanian populations of diverse malaria transmission intensity
Source: Front Immunol. 2025 Sep 11;16:1589061. doi: 10.3389/fimmu.2025.1589061 (PMC12460233; doi:10.3389/fimmu.2025.1589061)
Supplement: Supplementary file 2 [file Table2.docx]

**Table S1: Demographic characteristics of participants** **assessed for persistence of Pfs230D1M and Pfs48/45 antibodies overtime in Bagamoyo district, Tanzania.**

| Age group | Low-moderate transmission | | |  | High transmission | | |
| --- | --- | --- | --- | --- | --- | --- | --- |
|  | Female | Male | ***Subtotal*** |  | Female | Male | ***Subtotal*** |
|  | n (%) | n (%) | ***n (%)*** |  | n (%) | n (%) | ***n (%)*** |
| Children | 4 (66.7) | 4 (33.3) | **8 (44.4)** |  | 11(40.7) | 5 (41.7) | **16 (41.0)** |
| Adult | 2 (33.3) | 8 (66.7) | **10 (55.6)** |  | 16 (59.3) | 7 (58.3) | **23 ((59.0))** |
| ***Subtotal*** | **6 (100)** | **12 (100)** | **18 (100)** |  | **27 (100)** | **12 (100)** | **39 (100)** |

**Table S2: TRA of Pfs230D1M and Pfs48/45 positive total IgG from semi-immune volunteers in Bagamoyo district, Tanzania.**

| SMFA-1 | Sample ID | Mean oocyst count | %TRA | 95%CI Low | 95%CI High | p-value |
| --- | --- | --- | --- | --- | --- | --- |
|  | Naive serum | 41.7 |  |  |  |  |
|  | 4B7 | 1.4 |  |  |  |  |
|  | N006*• | 13.2 | **60.7** | 15.4 | 81.8 | **0.022** |
|  | N008*• | 4.1 | **87.8** | 73.0 | 94.7 | **0.001** |
|  | N019*• | 31.2 | 7.0 | -96.9 | 57.4 | 0.792 |
|  | N001• | 32.4 | 3.6 | -101.6 | 54.7 | 0.918 |
|  | N021• | 36.7 | -9.2 | -137.5 | 50.2 | 0.796 |
|  | N026* | 32.3 | 3.9 | -102.8 | 57.4 | 0.911 |
|  | N055* | 33.3 | 0.9 | -113.6 | 55.8 | 0.935 |
|  | N027* | 23.0 | 31.4 | -50.6 | 68.0 | 0.316 |
|  | N028* | 26.5 | 21.2 | -63.9 | 63.1 | 0.531 |
|  | N036* | 34.3 | -2.1 | -120.1 | 52.7 | 0.935 |
| SMFA-2 | Naive serum | 74.0 |  |  |  |  |
|  | 4B7 | 3.0 |  |  |  |  |
|  | N006*• | 1.4 | **93.0** | 84.7 | 97.0 | **0.001** |
|  | N008*• | 0.1 | **99.7** | 99.1 | 99.9 | **0.001** |

*• = reactive to both Pfs48/45 and Pfs230D1M, • = reactive to Pfs48/45 only, * = reactive to Pfs230D1M only
